# The association between MTHFR 677C>T polymorphism and cervical cancer: evidence from a meta-analysis

**DOI:** 10.1186/1471-2407-12-467

**Published:** 2012-10-11

**Authors:** Qiang Mei, Daijun Zhou, Jinyu Gao, Shu Shen, Jinlin Wu, Linna Guo, Zhiqing Liang

**Affiliations:** 1 4th team of Cadet Brigade, the Third Military Medical Univrersity, Chongqing, 400038, China; 2 Department of Gynecology and Obstetrics, Huai’an First People’s Hospital, Nanjing Medical University, Huai'an, Jiangsu province, 223300, China; 3 Class 2 of Grade 2008West China Medical School of Sichuan University, Chengdu, Sichuan province, 610041, China; 4 Department of Endocrinology and Metabolism, the Second Affiliated Hospital of Chongqing Medical University, Chongqing, 400010, China; 5 Department of Internal Medicine, the Second Affiliated Hospital of Tianjin Medical University, Tianjin, 300052, China; 6 Department of Obstetrics and Gynecology, Southwest Hospital of the Third Military Medical University, Chongqing, 400038, China

**Keywords:** MTHFR, Single nucleotide polymorphism, Cervical cancer, Meta-analysis

## Abstract

**Background:**

MTHFR 677C>T polymorphism is a genetic alteration in an enzyme involved in folate metabolism, but its effect on host susceptibility to cervical cancer is still uncertain. The aim of this study was to investigate the association between MTHFR 677C>T polymorphism and cervical cancer by performing a meta-analysis.

**Methods:**

Pubmed, Embase, Web of Science, and the Chinese Biomedical Database (CBM) databases were searched for case–control studies investigating the association between MTHFR 677C>T polymorphism and cervical cancer. Odds ratios (OR) and 95% confidence intervals (95%CI) were used to assess this possible association.

**Results:**

11 studies with a total of 1898 cervical cancer cases and 2678 controls were included. Meta-analyses of a total 11 studies showed no association between MTHFR 677C>T polymorphism and cervical cancer using all five genetic models (All P values > 0.05). However, subgroup analyses showed the odds of the homozygous TT genotype were much less in cervical cancer cases than in controls in Europeans, which implied an association between the homozygous TT genotype and cervical cancer in Europeans (For TT versus CC, fixed-effects OR = 0.65, 95%CI 0.45-0.93, P = 0.020, I^2^ = 0.0%). The odds for the homozygous TT genotype were greater in cervical cancer cases than in controls in East Asians, which also implied an association between the homozygous TT genotype and cervical cancer in East Asians (For TT versus CC, random-effects OR = 1.66, 95%CI 1.05-2.62, P = 0.029, I^2^ = 52.6%; For TT versus CT/CC, random-effects OR = 1.55, 95%CI 1.09-2.22, P = 0.016, I^2^ = 42.4%). Both subgroup analyses and meta-regression analyses suggested ethnicity was the major source of heterogeneity. Publication bias was not evident.

**Conclusions:**

This meta-analysis supports an association between MTHFR 677C>T polymorphism and cervical cancer, and the effect of this association may be race specific. Further studies with large sample sizes and careful design are needed to identify this association more comprehensively.

## Background

Cervical cancer is the third most commonly diagnosed cancer and the fourth leading cause of cancer death in women worldwide [1]. Global cervical cancer incidence increased from 378,000 cases per year in 1980 to 454,000 cases per year in 2010, and killed 200,000 women in 2010. Of these, 46,000 were found in developing countries in the 15–49 year old age bracket [2]. Though there have been many advances in the classification, diagnosis and treatment of cervical cancer, it is still a public health concern [2]. The mechanism of cervical carcinogenesis remains unclear, and multiple environmental and lifestyle factors may increase the risk of developing cervical cancer, including human papilloma virus (HPV) [3,4]. However, not all of those subjects exposed to these risk factors develop cervical cancer, which suggests genetic factors may also play an important role in the host's susceptibility to the disease.

Methylenetetrahydrofolate reductase (MTHFR) converts 5, 10-methylenetetrahydrofolate to 5-methyltetrahydrofolate and directs the homeostasis between DNA synthesis and methylation [5]. The MTHFR 677C>T (rs1801133) polymorphism changes the amino acid 222 from Alanine to Valine and decreases the MTHFR enzyme activity [6,7]. Individuals with the MTHFR 677C>T homozygous TT have only 25% of MTHFR enzyme activity, which reduces plasma folate levels and elevates plasma homocysteine levels. This homozygous TT genotype may confer elevated plasma homocysteine levels, lifelong DNA hypomethylation, and increased risk of cancer [8,9]. However, the homozygous TT genotype also can cause a greater availability of 5,10-methylenetetrahydrofolate and thymidine, increasing purine synthesis and leading to smaller probability of DNA strand breakage [10,11]. Thus, the MTHFR 677C>T TT genotype may therefore confer both an increased as well as decreased risk of cancer [10,11]. Many case–control studies have been published to assess the association between MTHFR 677C>T polymorphism and cervical cancer, but the available evidence for this genetic association is still weak, owing to disagreements among the conclusions from those studies [12-18]. Small genetic association studies have various designs, different methodologies and insufficient power, and fail to demonstrate a strong correlation, while combining data from all eligible studies by meta-analysis has the advantage of reducing random error and obtaining precise estimates for potential genetic associations [19,20]. To shed some light on this possible association between MTHFR 677C>T polymorphism and cervical cancer, we presented herein the results of a meta-analysis of published data. We followed the Meta-analysis of Observational Studies in Epidemiology (MOOSE) consensus statement during stages of design, implementation, and reporting of this meta-analysis [21].

## Methods

### Search strategy

We conducted a comprehensive search in the Pubmed, Embase, Web of Science, and Chinese Biomedical Database (CBM) databases from their inception through August 22, 2011 and updated on May 6, 2012. We combined search terms for MTHFR 677C>T polymorphism and cervical cancer. Search terms included: (Methylenetetrahydrofolate reductase, MTHFR, C677T, 677C>T, or rs1801133); and (cervical carcinoma, cervical cancer, cervical tumor, or cervix cancer). There was no language limitation. All references cited in the included studies were also reviewed to identify additional published articles not indexed in common databases.

### Study eligibility

Eligibility criteria included the following: 1) case–control design with genotyping of individuals with and without cervical cancer 2) cervical cancer confirmed histologically or pathologically 3) sufficient reported genotypic frequencies in both cases and controls for estimating an odds ratio (OR) with a 95% confidence interval (95%CI) 4) the genotype distribution among the control population consistent with Hardy-Weinberg Equilibrium (HWE). In studies with overlapping cases or controls, only the largest study with extractable data was included in the meta-analysis. Studies without relevant data or about cervical intraepithelial neoplasia (CIN) were excluded. Studies investigating cancer progression, severity, phenotype modification, response to treatment, or survival were excluded from this review. Family-based association studies were also excluded due to differing study designs.

### Data extraction

Two investigators independently extracted data, and disagreements were resolved through consensus. Standardized abstraction sheets were employed for recording data from individual studies. Data retrieved from these studies included the following: author, year of publication, study design, study population, ethnicity of the study population (categorized as Europeans, East Asians, Indians, and others), demographics, genotyping method, adjustment for known confounding variables (age, smoking, alcohol consumption, folate, HPV status, etc.), and the genotype distribution of MTHFR 677C>T polymorphism in the cases and controls.

### Quality assessment

Quality assessment of case–control studies in this meta-analysis was performed using the Newcastle Ottawa scale (NOS) as recommended by the Cochrane Non-Randomized Studies Methods Working Group [22-24]. This instrument was developed to assess the quality of nonrandomized studies, specifically cohort and case–control studies. Based on the NOS, case–control studies were judged based on three broad perspectives: selection of study groups (1 criterion), comparability of study groups (4 criteria), and ascertainment of outcome of interest (3 criteria). Given the variability in quality of observational studies found on our initial literature search, we considered studies that met 5 or more of the NOS criteria as high quality [22-24].

### Statistical analysis

We performed a meta-analysis of the association between MTHFR 677C>T polymorphism and cervical cancer under the allele contrast (T versus C), homozygote (TT versus CC), heterozygote (CT versus CC), recessive (TT versus CT and CC), and dominant (TT and CT versus CC) models. We calculated the pooled OR with its corresponding 95%CI to assess this possible association. The significance of the pooled OR was determined by the Z test, and a P value of less than 0.05 was considered significant. In our study, two models of meta-analysis for dichotomous outcomes were utilized: the random-effects model and the fixed-effects model [25,26]. The random-effects model was performed, using the DerSimonian and Laird’s method [26], while the fixed-effects model was conducted using the Mantel-Haenszel’s method [25]. To assess the between-study heterogeneity more precisely, both the chi-square based Q statistic test and the I^2^ statistic were calculated [27,28]. I^2^ values of 25%, 50%, and 75% were used as evidence of low, moderate, and high heterogeneity, respectively [27]. The random-effects model was used to pool the data when moderate heterogeneity existed; however, the data were not pooled if high heterogeneity existed (I^2^ value > 75%). In addition, the fixed-effects model was used to pool the data when low heterogeneity existed (I^2^ value < 50%). For additional analyses, the cases and controls were subgrouped by ethnicity. Ethnicity was firstly categorized into Caucasians, East Asians, Africans, and others according to racial classifications for genetic studies [30,31]. Caucasians were further categorized into Europeans and Indians [30,31]. East Asians mainly included China, Korea, and Japan. Because characteristics of participants were not consistent between studies, we further conducted meta-regression analysis to explore possible explanations for heterogeneity [29]. To validate the credibility of outcomes in this meta-analysis, sensitivity analysis was performed by sequential omission of individual studies or by omitting studies without high quality [32]. Potential publication bias was assessed by visual inspection of the Begg’s funnel plot, and a symmetric plot suggested low risk of possible publication bias [33]. In addition, we also performed the Egger linear regression test at the P < 0.10 level of significance to assess the publication bias [34]. All analyses were performed using STATA version 12.0 (StataCorp LP, College Station, Texas). A P value < 0.05 was considered statistically significant, except where specified.

## Results

### Characteristics of included studies

**Figure 1:**
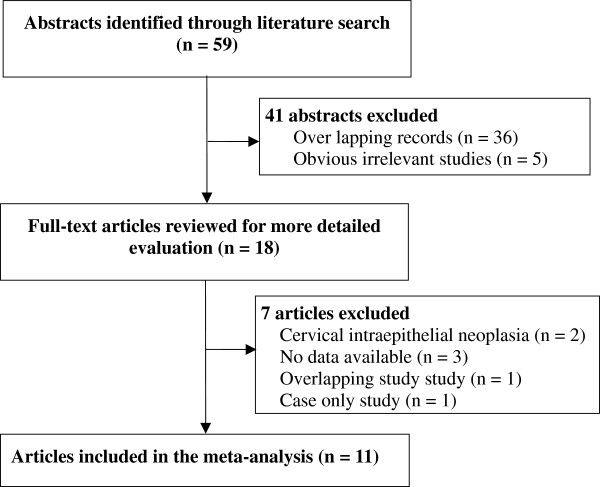
**Flow chart of study selection in the meta-analysis of MTHFR 677C>T polymorphism with cervical cancer.**

A flow diagram illustrating the study selection process is shown in Figure 1. With our original search criterion, 59 abstracts were found. After discarding those which did not meet the criteria clearly and excluding 41 records, 18 publications were preliminarily identified for further detailed evaluation [12-18,35-45]. After reviewing each original paper and extracting data, 7 publications were excluded including two studies on CIN [36,41], three for lack of available data [37-39], one for case-only study [35] and one for overlapping study [40]. Following these exclusions, 11 individual case–control studies with a total of 1, 898 cases and 2, 678 controls were included into this meta-analysis [12-18,42-45]. 

Table 1 presents a brief description of these 11 case–control studies. Ethnic groups among these studies were as following: 6 Caucasians, 4 East Asians and one from the Mexican population. There were 3 studies in Europeans [18,14, and 43] and 3 studies in Indians [13,15, and 16]. All 11 studies were hospital-based case–control studies. Five studies selected controls from healthy individuals, five selected controls from non-cancer individuals, and one selected controls from non-cancer patients with hysteromyoma (Table 1). The confounding factors were reported in 7 studies, and age was the most common confounding factor (Table 1). The number of cases varied from 21 to 636, and the number of controls varied from 74 to 592 (Table 1). The distribution of the MTHFR 677C>T genotype in the control groups of these 11 studies was all consistent with HWE (All P _HWE_ values were more than 0.05, Table 1). According to the quality criteria, there were 10 studies of high quality, and one low quality (Table 1). 

**Table 1:** **Characteristics of studies on the association between MTHFR 677**C>T **polymorphism and cervical cancer**

**Author (year)**	**Ethnicity**	**Country**	**Study design**	**Cases**	**Controls**	**Genotype method†**	**Genotype frequency (TT:CT:CC)**	**Confounding factors**	**P**_HWE_*	**Quality score**
Lambropoulos 2003 [43]	Europeans	Greece	Hospital-based case–control study	21 cases with cervical cancer	91 non-cancer controls	PCR-RFLP	(case) 2:8:11 (control) 12:37:42	None	0.40	5
Sull 2004 [45]	East Asians	South Korea	Hospital-based case–control study	246 cases with invasive cervical cancer	454 healthy controls	PCR-RFLP	(case) 58:115:73 (control) 80:221:153	Age, weight	0.99	7
Zoodsma 2005 [18]	Europeans	Netherlands	Hospital-based case–control study	636 cases with cervical cancer	592 unrelated controls	PCR-RFLP	(case) 49:230:357 (control) 57:262:273	None	0.61	6
Kang 2005 [42]	East Asians	South Korea	Hospital-based case–control study	79 cases with invasive cervical cancer	74 healthy controls	PCR-RFLP	(case) 20:32:27 (control) 12:32:30	Age	0.49	7
Delgado 2006 [12]	Others	Mexico	Hospital-based case–control study	70 cases with cervical cancer	89 non-cancer controls	PCR-RFLP	(case) 18:34:18 (control) 20:49:20	None	0.34	5
Ma 2006 [44]	East Asians	China	Hospital-based case–control study	111 cases with cervical cancer	111 controls with hysteromyoma	PCR-RFLP	(case) 38:53:20 (control) 18:60:33	Age	0.29	4
Shekari 2008 [16]	Indians	India	Hospital-based case–control study	200 cases with cervical cancer	200 non-cancer controls	PCR-RFLP	(case) 2:28:170 (control) 7:68:125	Age, menopause	0.54	8
Kohaar 2010 [13]	Indians	India	Hospital-based case–control study	203 cases with cervical cancer	231 healthy controls	PCR-RFLP	(case) 4:58:141 (control) 5:65:161	Age	0.59	6
Prasad 2011 [15]	Indians	India	Hospital-based case–control study	62 cases with cervical cancer	241 non-cancer controls	PCR-RFLP	(case) 0:5:57 (control) 1:12:228	None	0.07	5
Mostowska 2011 [14]	Europeans	Poland	Hospital-based case–control study	124 cases with cervical cancer	168 healthy controls	PCR-RFLP	(case) 9:59:56 (control) 18:81:69	Parity	0.42	7
Tong 2011 [17]	East Asians	South Korea	Hospital-based case–control study	146 cases with advanced cervical cancer	427 unrelated healthy female volunteers	PCR-RFLP	(case) 28:65:53 (control) 77:198:152	Age	0.37	8

(†PCR-RFLP, Polymerase chain reaction restriction fragment length polymorphism; *P _HWE_ was for the P value of Hardy-Weinberg equilibrium.)

### Meta-analysis

Table 2 shows the results of the association between MTHFR 677C>T polymorphism and cervical cancer (Table 2). Meta-analyses of the 11 studies showed no association between MTHFR 677C>T polymorphism and cervical cancer under the five genetic models (All P values > 0.05, Table 2). Sensitivity analyses by sequential omission of individual studies or studies with low quality did not materially alter the overall pooled ORs. 

**Table 2:** **Odds ratios and heterogeneity results in the meta-analysis of the association between MTHFR 677**C>T **polymorphism and cervical cancer**

**Contrast model**	**Studies (participants)**	**Fixed effects model**		**Random effects model**		**P**_**H**_**‡**	**I**^**2**^**(%)**
		OR[95%CI]*	**P**_**OR**_	**OR[95%CI]**	**P**_**OR**_		
**Total studies**#							
TT versus CC	11(4,576)	1.05(0.85-1.30)	0.644	1.06(0.72-1.56)	0.773	0.008	58.2%
CT versus CC	11(4,576)	0.81(0.70-0.92)	0.002	0.86(0.67-1.11)	0.247	0.002	63.5%
TT + CT versus CC	11(4,576)	0.83(0.73-0.95)	0.005	0.90(0.68-1.20)	0.481	<0.001	73.3%
TT versus CT + CC	11(4,576)	1.14(0.94-1.38)	0.171	1.14(0.84-1.54)	0.412	0.052	45.1%
**Caucasians**							
T versus C	6(2,769)	0.74(0.65-0.84)	<0.001	0.74(0.54-1.01)	0.056	0.004	71.0%
TT versus CC	6(2,769)	0.63(0.45-0.88)	0.007	0.64(0.45-0.89)	0.009	0.794	0.0%
CT versus CC	6(2,769)	0.69(0.58-0.82)	<0.001	0.74(0.50-1.09)	0.122	0.003	71.7%
TT + CT versus CC	6(2,769)	0.68(0.58-0.80)	<0.001	0.71(0.49-1.04)	0.082	0.003	72.8%
TT versus CT + CC	6(2,769)	0.73(0.52-1.01)	0.056	0.73(0.53-1.02)	0.067	0.871	0.0%
**East Asians**							
T versus C	4(1,648)	1.24(1.07-1.44)	0.004	1.28(1.02-1.62)	0.034	0.092	53.5%
TT versus CC	4(1,648)	1.56(1.17-2.08)	0.003	1.66(1.05-2.62)	0.029	0.097	52.6%
CT versus CC	4(1,648)	1.08(0.85-1.37)	0.514	1.08(0.85-1.37)	0.518	0.754	0.0%
TT + CT versus CC	4(1,648)	1.20(0.96-1.50)	0.102	1.21(0.95-1.54)	0.120	0.340	10.6%
TT versus CT + CC	4(1,648)	1.51(1.17-1.94)	0.001	1.55(1.09-2.22)	0.016	0.157	42.4%
**Europeans**							
T versus C	3(1,632)	0.77(0.66-0.89)	0.001	0.77(0.66-0.89)	0.001	0.828	0.0%
TT versus CC	3(1,632)	0.65(0.45-0.93)	0.020	0.65(0.45-0.93)	0.020	0.991	0.0%
CT versus CC	3(1,632)	0.71(0.58-0.88)	0.002	0.71(0.58-0.88)	0.002	0.552	0.0%
TT + CT versus CC	3(1,632)	0.70(0.58-0.86)	<0.001	0.70(0.58-0.86)	<0.001	0.658	0.0%
TT versus CT + CC	3(1,632)	0.75(0.53-1.07)	0.113	0.75(0.53-1.07)	0.113	0.922	0.0%
**Indians**§							
TT versus CC	3(1,137)	0.51(0.20-1.30)	0.157	0.54(0.19-1.59)	0.260	0.321	12.0%
TT versus CT + CC	3(1,137)	0.57(0.22-1.46)	0.239	0.60(0.23-1.59)	0.305	0.471	0.0%

(*OR = Odds Ratio; 95%CI = 95% Confidence Interval; † random = random-effects model; fixed = fixed-effects model; ‡P_H_, the P value of heterogeneity; # the data was not pooled in the analysis of the allele contrast model (T versus C) owing to the high heterogeneity (I^2^ = 79.8%); § the data was not pooled in the analyses of the allele contrast model (T versus C, I^2^ = 87.7%), heterozygote (CT versus CC, I^2^ = 87.8%), and dominant (TT and CT versus CC, I^2^ = 88.5%) models owing to the high heterogeneity.)

**Figure 2:**
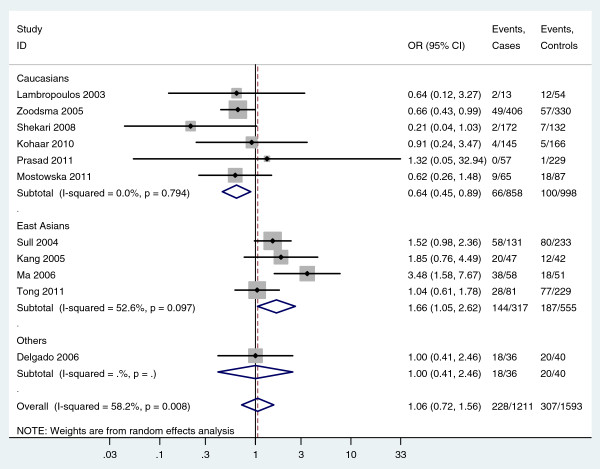
**Forest plot shows an association between the MTHFR 677C>T homozygous TT genotype and cervical cancer (TT versus CC).**

**Figure 3:**
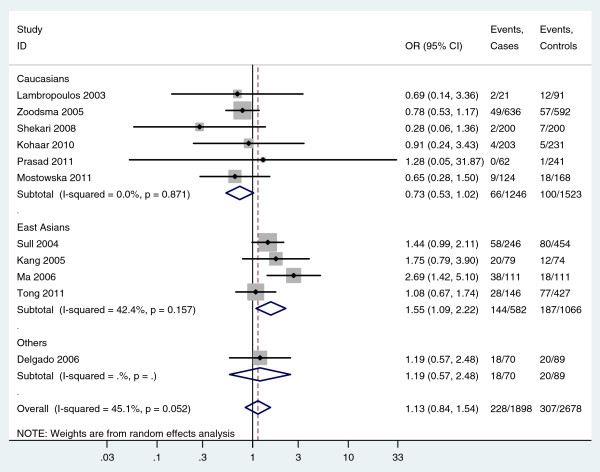
**Forest plot shows an association between the MTHFR 677C>T homozygous TT genotype and cervical cancer (TT versus CT/CC).**

In subgroup analyses, the odds of finding the homozygous TT genotype in subjects were much less in cases than controls in Caucasians, which implied an association between homozygous TT genotype and cervical cancer in Caucasians (For TT versus CC: fixed-effects OR = 0.63, 95%CI 0.45-0.88, P = 0.007; random-effects OR = 0.64, 95%CI 0.45-0.89, P = 0.009; I^2^ = 0.0%) (Figure 2, Table 2). However, the odds of finding the homozygous TT genotype in subjects was greater in cases than controls in East Asians, which also implied an association between the homozygous TT genotype and cervical cancer in East Asians (For TT versus CC: random-effects OR = 1.66, 95%CI 1.05-2.62, P = 0.029, I^2^ = 52.6%; For TT versus CT/CC: random-effects OR = 1.55, 95%CI 1.09-2.22, P = 0.016, I^2^ = 42.4%) (Figure 2, Figure 3, Table 2). For the subgroup analysis of East Asians, sensitivity analyses was further performed by excluding Ma et al.’s study which had low quality. Meta-analysis of the three remaining studies showed the odds of the homozygous TT genotype were still greater in cases than controls in East Asians (For TT versus CC: fixed-effects OR = 1.37, 95%CI 1.00-1.87, P = 0.050, I^2^ = 0.0%; For TT versus CT/CC: fixed-effects OR = 1.34, 95%CI 1.01-1.77, P = 0.039, I^2^ = 0.0%), which further indentified the association between the homozygous TT genotype and cervical cancer in East Asians. In addition, in the subgroup analysis of Europeans, there was no heterogeneity in any genetic models (All I^2^ = 0.0%, while the pooled results suggested an association between MTHFR 677C>T polymorphism and cervical cancer in Europeans under four genetic models (Table 2). However, no significant association was found in the subgroup analysis of Indians (Table 2). 

### Heterogeneity analysis

There was obvious heterogeneity in some genetic models for both the 11 studies and subgroup analyses (Table 2). Meta-regression analyses suggested ethnicity was the major source of heterogeneity in this meta-analysis under two comparison models (for TT versus CC, P = 0.048; for TT versus CT/CC, P = 0.045); however, no other sources were found by meta-regression analyses. Multivariate analyses of meta-regression were also performed, but no further definite source of heterogeneity were identified.

### Publication bias

**Figure 4:**
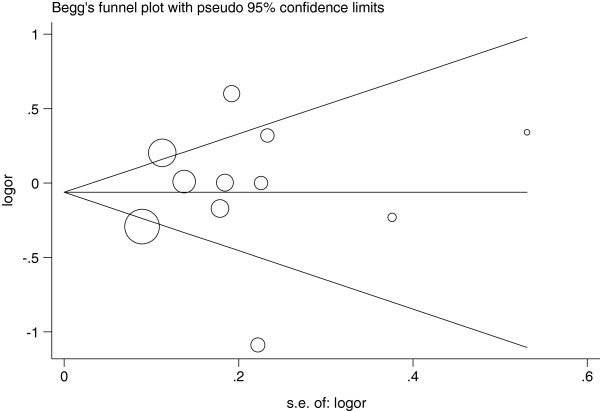
**Begg’s funnel plot for assessing the publication bias risk under the allele contrast model (T versus C, P **_**Egger**_ **= 0.744).**

Begg’s funnel plot and Egger’s test were used to assess the publication bias in this meta-analysis. The Funnel plots’ shape of all contrasts did not reveal obvious evidence of asymmetry, and all the P values of Egger’s test were more than 0.05, providing statistical evidence for the funnel plots’ symmetry. For example, in the meta-analysis investigating the association between MTHFR 677C>T polymorphism and cervical cancer under the allele contrast model (T versus C), the funnel plot’s shape was symmetrical, suggesting no presence of publication bias (Figure 4); in addition, the P value of the Egger’s test for the allele contrast model was 0.744, providing statistical evidence for funnel plot symmetry. Thus, the above results suggest that publication bias was not evident in this meta-analysis. 

## Discussion

Many case–control studies have been published to assess the association between MTHFR 677C>T polymorphism and cervical cancer, but the available evidence is still weak owing to disagreements among those studies [12-18]. Also, no meta-analysis assessing the association between MTHFR 677C>T polymorphism and cervical cancer has been performed. Thus, there is a need to perform a meta-analysis of published data investigating the association between MTHFR 677C>T polymorphism and cervical cancer to shed light on the contradictory findings. This meta-analysis investigating the association between MTHFR 677C>T polymorphism and cervical cancer was based on 11 studies, which gave a larger set of data for detecting significant differences. Meta-analyses of these 11 studies showed that there was no association between MTHFR 677C>T polymorphism and cervical cancer under any of the five genetic models (All P values > 0.05). However, subgroup analyses showed the odds of finding a homozygous TT genotype in subjects was much less in cases than controls in Europeans while the odds of finding the homozygous TT genotype was greater in cases than controls in East Asians, which implied an association between homozygous TT genotype and cervical cancer. Meta-regression analyses suggested ethnicity was the major source of heterogeneity in this meta-analysis. Publication bias was not evident in this meta-analysis. Thus, these findings support an association between MTHFR 677C>T polymorphism and cervical cancer, and there may be a race-specific effect in this association.

**Figure 5:**
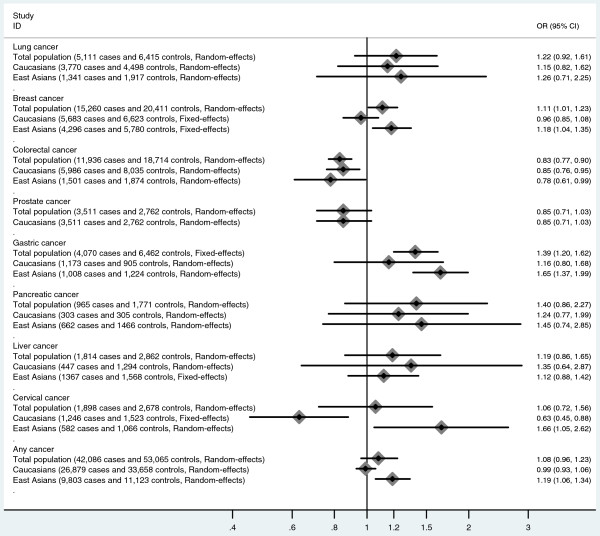
**Summary of the associations between MTHFR 677C>T polymorphism and different cancers (data were extracted from meta-analyses published previously [11,46-51] and present meta-analysis).**

Several large-scale meta-analyses combining data from multiple studies have been published investigating the association between MTHFR 677C>T polymorphism and various cancers such as gastric cancer, lung cancer, breast cancer, colorectal cancer, and liver cancer (Figure 5) [11,46-51]. Mazaki *et al.* and Jin et al. reported no association between liver and pancreatic cancers and MTHFR 677C>T polymorphism [47,49]. Zhang *et al.* reported the MTHFR 677C>T homozygous TT genotype may be associated with gastric cancer among Asians but not in Europeans [51]. However, Taioli *et al.* found that the MTHFR 677C>T homozygous TT genotype was associated with colorectal cancer among both Asians and Europeans [50]. Zacho *et al.* reported an association between MTHFR 677C>T the homozygous TT genotype and any site cancer in Asians but not in Europeans [11]. Our meta-analysis suggests the occurrence of the MTHFR 677C>T homozygous TT genotype is much less common in cases than controls in Europeans, but is greater in cases than controls in East Asians, which implies an association between MTHFR 677C>T polymorphism and cervical cancer. Thus, MTHFR 677C>T polymorphism may exert different effects in different kinds of cancer, and there might be race-specific effect in those associations. 

Heterogeneity is a very important part of meta-analysis, and finding the possible sources for the high heterogeneity is very important and can greatly affect the results of a meta-analysis [52]. To explore the possible sources for the high heterogeneity in present meta-analysis, we performed two steps including subgroup analysis and meta-regression analysis. By subgroup analysis and meta-regression analysis, we found ethnicity was the major source of the high heterogeneity in our meta-analysis, which could be explained by the race-specific effect of MTHFR 677C>T polymorphism on susceptibility to cervical cancer. However, ethnicity didn’t explain all heterogeneity in this meta-analysis, and no other sources were found by meta-regression analysis.

MTHFR regulates the metabolism of folate, and it is an important factor in DNA methylation and synthesis. Both heterozygous and homozygous variants of MTHFR 677C>T polymorphism have reduced MTHFR enzyme activity compared with the homozygous normal wild-type genotype. Reduction of MTHFR enzyme activity can increase the pool of 5, 10-methylene-THF at the expense of the pool of 5-methyl-THF and impair DNA methylation. Because DNA methylation plays a critical role in regulation of gene expression and maintenance of genomic stability, the aberrations in normal methylation patterns caused by MTHFR 677C>T polymorphism might further result in the development of cervical cancer by impairing the DNA methylation. Thus, there is also biological evidence for the association between MTHFR 677C>T polymorphism and cervical cancer. Our meta-analysis further provides epidemiological evidence for the association above. In addition, there is another common polymorphism in the MTHFR gene known as MTHFR 1298A>C polymorphism. MTHFR 1298A>C polymorphism is also associated with reduced levels of MTHFR enzyme and related to hyperhomocysteinemia, and it may also be associated with the development of cervical cancer [53]. However, there are only two case–control studies published to assess the association between MTHFR 1298A>C polymorphism and cervical cancer [40,42], and neither find an association. This lack of association may result from the limited sample size reflected in those two studies, and further studies with larger sample sizes are needed to identify this possible association.

Our analysis has several limitations that should be considered when interpreting the findings. Firstly, the main limitation of the meta-analysis was the inclusion of only a few studies with relatively small sample sizes and poor validation. There were only four studies from East Asian and three studies from Europeans. In addition, though we used two methods to assess the publication bias, these two methods may be limited in effectiveness for detecting the risk of publication bias, especially with the limited number of included studies. Thus, more studies with large sample sizes and careful design are needed to further identify this association more comprehensively. Secondly, our main analysis was based on unadjusted estimates owing to the lack of available data. However, a more precise analysis could be performed if adjusted estimates were available in all studies [54]. Furthermore, variability in the study designs and the selection of controls was revealed in present meta-analysis. Though we found that ethnicity was one major source of the high heterogeneity and could explain part of the high heterogeneity, no other sources were found. To get results from meta-analysis of homogeneous studies, more studies with homogeneously constructed design are needed in the future. Thirdly, the association between MTHFR 677C>T polymorphism and cervical cancer may be affected by the different histological types of cervical cancer. However, little data on this aspect were reported in the included studies, and we were unable to make subgroup analyses by histological type. Further studies are needed to identify this association in different histological types of cervical cancer. Finally, gene-gene and gene-environmental factors interactions were not fully addressed in this meta-analysis due to the lack of sufficient data. Previous study suggested an association between methylation of CpG sites in the HPV genome (particularly in L2 and L1) and diagnosis of CIN3, as compared to viral clearance, and the MTHFR gene may have a potential effect on cervical cancer by impairing DNA methylation not only in the host genes but also on the viral genes [55]. Thus, the association between MTHFR 677C>T polymorphism and cervical cancer could be affected by host HPV status, and there is high probability of gene-HPV interaction effects. In addition, several other gene polymorphisms are also associated with cervical cancer, and there may be gene-gene interactions [4,56]. Future studies may further assess the possible gene-gene and gene-environmental interactions in the association between MTHFR 677C>T polymorphism and cervical cancer.

## Conclusions

In conclusion, our meta-analysis supports an association between MTHFR 677C>T polymorphism and cervical cancer, and there may be a race-specific effect in this association. In addition, further studies with larger sample sizes and careful design are needed to identify this association more comprehensively.

## Competing interests

None of the authors have any conflict of interests to declare.

## Authors’ contributions

ZD, MQ and GJ carried out the meta-analysis study, drafted the manuscript and involved in revising the manuscript critically for important intellectual content. SS and WJ participated in the design of the study and revised the manuscript. GL carried out the meta-analysis study and drafted the manuscript. LZ participated in the design of the study, drafted the manuscript and revised the manuscript. All authors read and approve the final manuscript.

## Funding

No external funding was either sought or obtained for this study.

## Pre-publication history

The pre-publication history for this paper can be accessed here:

http://www.biomedcentral.com/1471-2407/12/467/prepub

